# Single-Pill Combination Therapy of Amlodipine, Telmisartan, and Chlorthalidone in the Management of Hypertension: A Systematic Review of Randomized Controlled Trials

**DOI:** 10.7759/cureus.68802

**Published:** 2024-09-06

**Authors:** Shereef Elbardisy, Muteb N Alotaibi, Abdulbadih R Saad, Mshal Alhatm, Othman H Alharbi, Fajer B Alyaqout, Omar E Elshaer, Abdulaziz M Alazmi, Navyamani V Kagita, Ibrahim M Allam, Aaima I Bhutta, Shady Habboush, Raghad Sindi, Yasein Aswad, Abdullah M Alharran

**Affiliations:** 1 Department of Cardiology, Saudi German Hospital, Dubai, ARE; 2 College of Medicine, Alfaisal University, Riyadh, SAU; 3 College of Medicine and Medical Sciences, Arabian Gulf University, Manama, BHR; 4 Department of Endocrinology, Thomas Jefferson University Hospital, Philadelphia, USA; 5 Department of Medicine, Primary Health Care Corporation, Doha, QAT; 6 College of Medicine, Fatima Memorial Hospital, Lahore, PAK; 7 Faculty of Pharmacy, Umm Al-Qura University, Makkah, SAU

**Keywords:** amlodipine, blood pressure, chlorthalidone, hypertension, randomized control trials, systematic review, telmisartan

## Abstract

Hypertension is a major cause of cardiovascular disease and death worldwide. Low-dose combination therapy is a promising approach for managing hypertension due to its safety and efficacy. This systematic review evaluates the safety and efficacy of a single-pill, low-dose combination of amlodipine, telmisartan, and chlorthalidone for essential hypertension based on evidence from randomized controlled trials (RCTs). We followed the Preferred Reporting Items for Systematic Reviews and Meta-Analyses guidelines and searched the Cochrane, Scopus, PubMed, and Web of Science databases until July 01, 2024, using the following search string: (telmisartan) AND (amlodipine) AND (chlorthalidone) AND (randomized OR randomly). The quality of the RCTs was assessed using the revised Cochrane risk of bias tool. The primary endpoint was the mean change in sitting systolic blood pressure (BP), with secondary endpoints including BP target achievement rates, BP response rates, and serious treatment-related adverse events. Overall, three RCTs met the inclusion criteria and exhibited a low risk of bias. The doses in the combination pill ranged from 2.5 to 5 mg of amlodipine, 20 to 80 mg of telmisartan, and 4.167 to 25 mg of chlorthalidone. Control groups varied, including usual care, amlodipine 10 mg, and dual therapy of telmisartan and amlodipine. Results showed significant reductions in mean sitting systolic and diastolic BP, improved BP control and response rates, and a generally safe profile with no significant differences in serious adverse events. Despite encouraging data, results should be interpreted with caution due to heterogeneity in doses and control groups. Further research should address the long-term effects and explore predictors of response to this therapy.

## Introduction and background

Hypertension is a widespread condition that affects many people globally. According to recent data from an extensive study, the prevalence of hypertension among individuals aged 30 to 79 has significantly increased over the years. Specifically, the number of affected women rose from 331 million in 1990 to 626 million in 2019 [1]. Similarly, the number of men with hypertension grew from 317 million in 1990 to 652 million in 2019 [1]. These figures illustrate a dramatic doubling in the prevalence of hypertension over this period.

Hypertension exerts a significant impact on cardiovascular health. From a pathophysiological perspective, hypertension strains the arteries, making them stiffer and less elastic, which can lead to damage and narrowing over time. This increased pressure forces the heart to work harder, potentially resulting in heart failure or an enlarged heart. Additionally, hypertension accelerates the buildup of plaque in the arteries, raising the risk of coronary artery disease, heart attack, and stroke [2,3].

Hypertension is the leading global cause of cardiovascular disease and death. Specifically, it places a significant burden on countries with low- to middle-income levels [4]. In high-income countries, only about half of hypertension cases are properly treated and achieve target blood pressure (BP) levels. This proportion drops to a quarter in lower-income countries [5]. The primary reason for failing to reach target BP is the ongoing use of monotherapy, which often results in low efficacy [6]. Several international guidelines recommend that high-risk patients with hypertension should aim for lower BP targets, highlighting the need for improved treatment methods [7-9].

To fulfill its mission of reducing the global burden of high BP, the International Society of Hypertension has established international recommendations for treating hypertension in adults aged ≥18 years old [10]. Accumulating evidence from high-quality studies demonstrates that combination therapy using ≥2 antihypertensive drugs with dissimilar modes of action is likely to augment BP reduction and reduce the side effects associated with higher doses of a single drug [11-14]. Currently, low-dose combination therapy is an encouraging and effective choice for the primary therapy of hypertension, offering both safety and efficacy [15]. Based on guidelines from the United States [9] and Europe [16], the two most frequently used combinations for the management of hypertension comprise angiotensin-converting enzyme inhibitor (ACEI) or angiotensin receptor blocker (ARB) plus calcium channel blocker (CCB). A thiazide-like diuretic may be included as part of a triple combination therapy for patients with uncontrolled hypertension [9,10,16].

Mechanistically, ACEI (e.g., enalapril) works by blocking the conversion of angiotensin-I to angiotensin-II, a peptide that causes blood vessels to constrict, thereby lowering BP. ARB (e.g., telmisartan) prevents angiotensin-II from binding to its receptors on blood vessels, which also leads to vasodilation and reduced BP. CCB (e.g., amlodipine) inhibits calcium entry into the heart and vascular smooth muscle cells, causing these cells to relax and dilate blood vessels, which lowers BP and decreases heart workload. Thiazide-like diuretics (e.g., chlorthalidone) promote the excretion of sodium and water by the kidneys, reducing blood volume and consequently lowering BP. Together, these medications help manage hypertension through different but complementary mechanisms [9,10,16].

According to the TRIUMPH study (Triple Pill vs. Usual Care Management for Patients with Mild-to-Moderate Hypertension), patients who received the low-dose triple combination therapy (telmisartan/amlodipine/chlorthalidone) exhibited significantly lower systolic (-9.8 mmHg) and diastolic (-5.0 mmHg) BP compared to those who received standard care only. Additionally, there was no meaningful variation in the occurrence of side effects between the triple combination arm and the standard care group (6.6% vs. 6.8%) [17]. Since then, two randomized controlled trials (RCTs) have been conducted [18,19]. However, no systematic review and/or meta-analysis has been compiled to summarize the literature and provide concrete conclusions on the subject.

Therefore, we undertook this investigation to systematically review the existing evidence and possibly perform a quantitative meta-analysis. Our goal was to evaluate the safety and efficacy of the low-dose combination of amlodipine, telmisartan, and chlorthalidone in managing patients with essential hypertension.

## Review

Methodology

As this research involved the analysis of published literature, formal ethical approval was not required. This study adhered to the guidelines outlined in the Preferred Reporting Items for Systematic Reviews and Meta-Analyses (PRISMA) statement [20].

The inclusion criteria were as follows: (i) patients diagnosed with hypertension; (ii) the experimental group received triple therapy of telmisartan, amlodipine, and chlorthalidone; (iii) the control group received standard care (amlodipine or dual therapy of amlodipine and telmisartan); (iv) reliable reporting of mean change in sitting systolic BP; and (v) the study design was RCT. Studies that did not meet all of these inclusion criteria were excluded from our review.

From inception until July 01, 2024, we searched the Cochrane, Scopus, PubMed, and Web of Science databases using the following search string: (telmisartan) AND (amlodipine) AND (chlorthalidone) AND (randomized OR randomized OR randomly). We did not apply filters during the screening of data sources. The screening process and study selection underwent three steps. First, duplicate citations were deleted. Second, titles/abstracts were reviewed. Third, full texts were assessed to make the final decision on inclusion in the review. To reduce the risk of overlooking citations, we meticulously examined the references of all reviewed RCTs and contemporary reviews manually. Two authors independently completed the database screening and study selection, and disputes were rectified through discussion with the leading author.

For data extraction, we gathered information on the characteristics of the reviewed RCTs and their research subjects, including the author’s name, publication date, country, study groups, drug doses, number of patients, age, gender, body mass index, and prevalence rates of diabetes mellitus (DM) and chronic kidney disease (CKD). The primary endpoint in this review was the mean change in sitting systolic BP as reported in the RCTs. The secondary endpoints included the rate of patients achieving the target BP, the rate of BP response, and the rate of safety outcomes as reported in the RCTs. The target BP achievement criteria were as follows: for patients without DM or CKD, it was mean sitting systolic BP <140/90 mmHg. For patients with DM or CKD, the target BP was a mean sitting systolic BP <130/80 mmHg. The BP response was defined as a reduction in mean sitting systolic BP of ≥20 mmHg and/or mean sitting diastolic BP of ≥10 mmHg from baseline values. The safety outcomes included the rates of serious treatment-related adverse events. The quality of each included RCT was evaluated according to the Cochrane risk of bias tool, version 2 [21]. Data collection involved three pairs of co-authors, with any conflicts resolved through dialogue with the leading author.

Due to the variation in control groups and the differences in triple therapy doses across all studies, conducting a meta-analysis was not technically feasible. Therefore, we qualitatively summarized the findings of the studies.

Results

**Figure 1:**
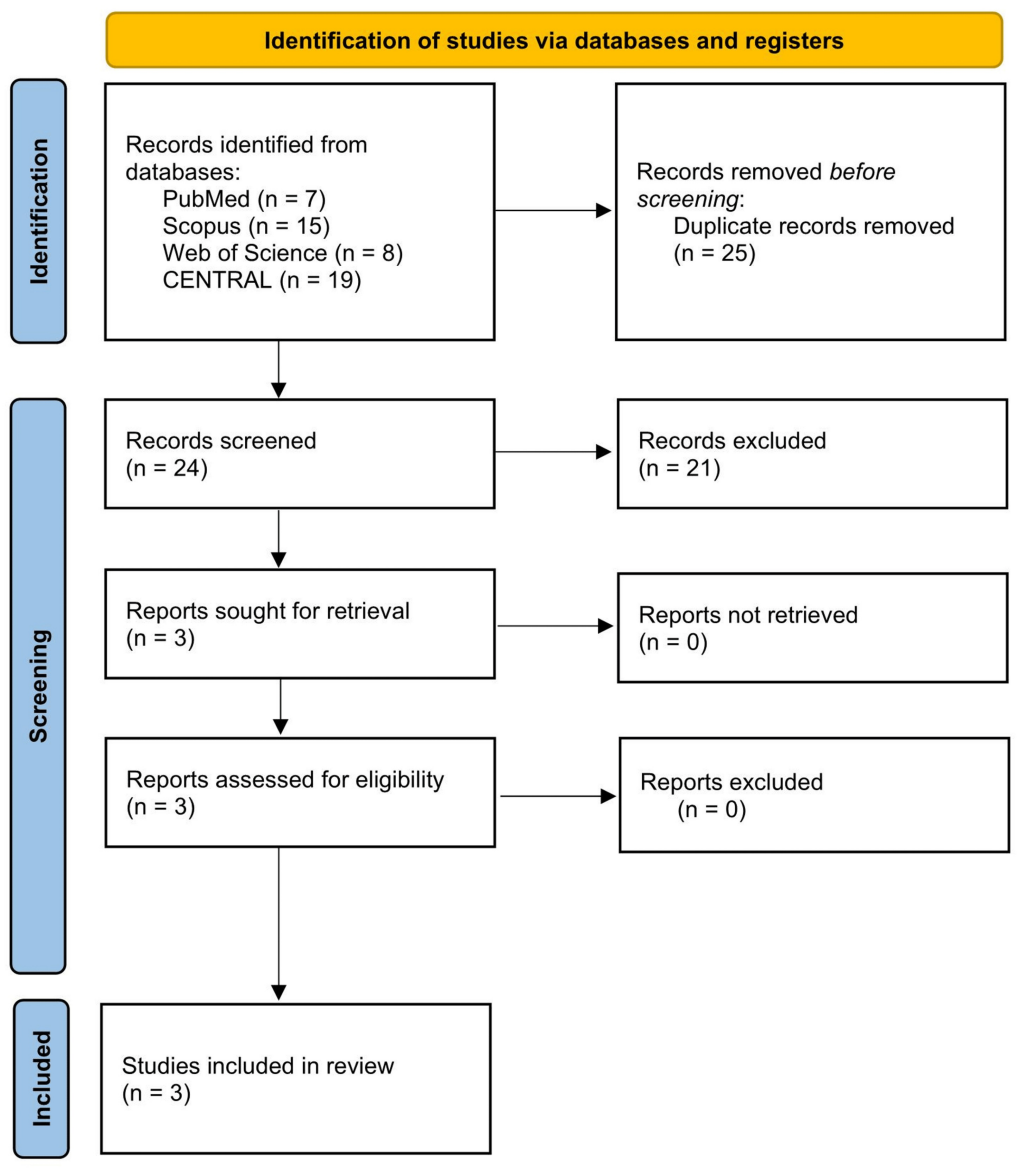
Summary of the PRISMA flowchart and the study selection process. PRISMA: Preferred Reporting Items for Systematic Reviews and Meta-Analyses

Figure 1 provides an overview of the PRISMA flowchart and the study selection process. Three RCTs met the inclusion criteria and were included in the review [17-19].

Table 1 summarizes the characteristics of these RCTs, which were conducted in Sri Lanka (n = 1) and Korea (n = 2) and published between 2018 and 2023. These studies involved patients with essential hypertension. In the triple therapy pill, the doses of amlodipine ranged from 2.5 to 5 mg, telmisartan from 20 to 80 mg, and chlorthalidone from 4.167 to 25 mg. The control groups varied and included usual care (with clear specification), amlodipine 10 mg, and a dual therapy of telmisartan 40 mg and amlodipine 5 mg (later increased to telmisartan 80 mg and amlodipine 5 mg).

**Table 1:** The baseline characteristics of the included studies. Categorical variables are presented as numbers (percentages), whereas numerical variables are presented as means ± standard deviations. AML: amlodipine; BMI: body mass index; CHTD: chlorthalidone; CKD: chronic kidney disease; DBP: diastolic blood pressure; DM: diabetes mellitus; n: sample size; SBP: systolic blood pressure; TEL: telmisartan; yr: year

Study	Country	Phase	Duration	Arms	Drugs and doses	n	Age (yr)	Female	BMI (kg/m^2^)	Smoking	DM	CKD	Baseline SBP (mmHg)	Baseline DBP (mmHg)
Webster et al., 2018 [17]	Sri Lanka	III	6 months	Triple pill	AML 2.5 mg + TEL 20 mg + CHTD 12.5	349	56.4 ± 11.3	207 (59.3)	Obese, 64 (18.3)	39 (11.2)	112 (32.3)	7 (2)	154.2 ± 11.3	89.5 ± 9.7
				Control	Usual care	351	56 ± 10.7	196 (55.8)	Obese, 68 (19.4)	34 (9.7)	109 (30.6)	3 (0.9)	154.2 ± 11.6	90 ± 9.7
Sung et al., 2022 [19]	Korea	II	8 weeks	Triple pill	AML 2.5 mg +TEL 20 mg + CHTD 12.5	25	64.16 ± 6.68	10 (40)	25.80 ± 4.18	17 (68)	25 (100)	0 (0)	153.17 ± 10.39	91.96 ± 8.84
				Control	AML 10 mg	25	59.88 ± 10.82	8 (32)	27.02 ± 3.62	14 (56)	25 (100)	0 (0)	149.87 ± 12.89	90.80 ± 10.12
Cho et al., 2023 [18]	Korea	III	8 weeks	Triple pill	AML 5 mg + TEL 40 mg + CHTD 12.5 for 2 weeks, followed by AML 5 mg + TEL 80 mg + CHTD 25 for 6 weeks	186	61.5 ± 10.6	42 (22.6)	26.2 ± 3.5	Not reported	39 (21)	26 (14)	149.9 ± 12.2	88.5 ± 10.4
				Control	AML 5 mg + TEL 40 mg for 2 weeks, followed by AML 5 mg + TEL 80 mg for 6 weeks	188	60.3 ± 10.8	39 (20.7)	26.7 ± 3.5	Not reported	51 (27.1)	28 (14.9)	149.9 ± 12.2	88.5 ± 10.4

**Figure 2:**
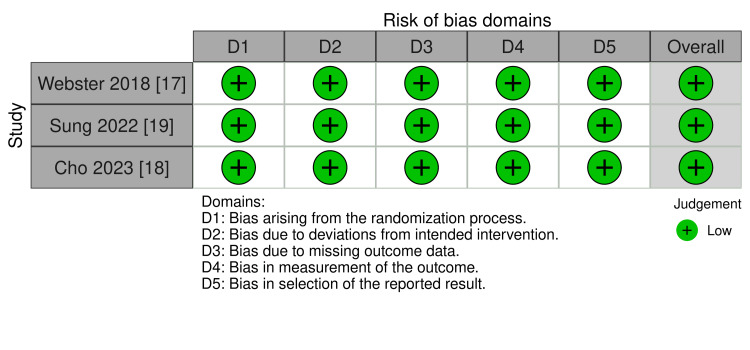
Summary of the risk of bias (quality) of the included studies.

The studies included patients of both genders, with nearly a quarter having DM. All RCTs had an overall low risk of bias (Figure 2).

In 2018, Webster et al. conducted a phase III study in Sri Lanka to gauge the safety and efficacy of a low-dose triple combination pill (2.5 mg of amlodipine, 20 mg of telmisartan, and 12.5 mg of chlorthalidone) contrasted to standard care [17]. A total of 700 adult individuals with essential hypertension were recruited, with 349 patients allocated to the triple therapy arm and 351 to the standard care arm. The average age of the entire cohort was 56 years. Overall, 58% of the cohort was women. Moreover, 29% of the participants had diabetes. The mean baseline systolic/diastolic BP was 154/90 mmHg. The primary endpoint was achieving the target BP at six months, which was substantially higher in the triple therapy arm contrasted to the standard care arm (69.5% vs. 55.3%, p < 0.001). For the secondary outcomes, the rates of achieving the BP target at six weeks (67.8% vs. 43.6%, p < 0.001) and 12 weeks (72.6% vs. 47.4%, p < 0.001) were also substantially higher in the triple therapy arm contrasted to the standard care arm. Moreover, at six months, the triple therapy arm displayed substantial reductions in systolic BP (mean difference = -8.8 mmHg; 95% confidence interval (CI) = -11.2, -6.4; p<0.001) and diastolic BP (mean difference = -4.6 mmHg; 95% CI = -6, -3.1; p < 0.001) contrasted to the standard care arm. There was no variation in the self-reported compliance of medication use at six months (95% vs. 94.6%, p = 0.82) or the frequency of withdrawal due to adverse events (6.6% vs. 6.8%, p = 0.92). Regarding adverse events, 38.1% of triple therapy patients had ≥1 adverse event contrasted to 34.8% in the standard care arm. The top three most frequent adverse events comprised musculoskeletal pain, dizziness and related syncope, and headache. The study concluded that for patients with essential hypertension, treatment with a low-dose triple combination pill led to a higher fraction of patients attaining their target BP contrasted to standard care. This approach, either as initial therapy or as a replacement for a single therapy, may be a successful strategy for improving BP control.

In 2022, Sung et al. conducted a phase II trial in Korea to investigate the therapeutic value and safety of a half-dose triple therapy pill (chlorthalidone 6.25 mg, telmisartan 20 mg, and amlodipine 2.5 mg) compared to a high dose of amlodipine (10 mg) over eight weeks [19]. The study included 50 adult patients with essential hypertension, equally divided into two arms of 25 patients each. The average age of the entire cohort was roughly 62 years, with 64% being women, 24% having diabetes, and a mean baseline systolic/diastolic BP of 151.5/91.4 mmHg. The primary endpoint was the change in mean sitting systolic BP at eight weeks, showing no notable variation between the two arms: the triple therapy arm had a mean difference of −19.55 ± 14.75 mmHg, while the amlodipine 10 mg group had a mean difference of −17.07 ± 13.92 mmHg (p=0.9783). For secondary endpoints, no substantial change in mean sitting diastolic BP was observed between the two arms at four weeks (−18.63 ± 13.73 mmHg vs. −15.00 ± 11.77 mmHg, p = 0.5569). The percentage of patients who achieved BP control at four weeks (58.33% vs. 45.83%) and eight weeks (58.33% vs. 52%) did not significantly vary between the arms. Similarly, the percentage of patients achieving a BP response did not significantly differ at four weeks (41.67% vs. 12.5%) and eight weeks (45.83% vs. 24%). No serious adverse drug reactions were registered in either arm. These findings indicate that an eight-week regimen of half-dose amlodipine, telmisartan, and chlorthalidone did not significantly differ in BP control compared to high-dose amlodipine in patients with essential hypertension, and it did not raise any safety concerns.

In 2023, Cho et al. [18] conducted a phase III study in Korea to explore the clinical utility and safety of a triple therapy pill (amlodipine 2.5 mg, telmisartan 40 mg, and chlorthalidone 12.5 mg) compared to dual therapy (amlodipine 2.5 mg and telmisartan 40 mg) for two weeks. This was followed by an escalation to a higher dose of triple therapy (amlodipine 5 mg, telmisartan 80 mg, and chlorthalidone 25 mg) versus dual therapy (amlodipine 5 mg and telmisartan 80 mg) for six weeks. A total of 381 adult individuals with essential hypertension were recruited, with 186 patients appointed to the triple therapy arm and 188 to the dual therapy arm. The mean age of the cohort was 61 years, 21.8% were women, 24.1% had diabetes, and the mean baseline systolic/diastolic BP was 149.9/88.5 mmHg. The primary endpoint was the change in mean sitting systolic BP at eight weeks, where the triple therapy arm showed significant reductions (mean difference = −7.5 ± 1.5, p < 0.0001). For secondary endpoints, the triple therapy arm also displayed substantial reductions in diastolic BP (mean difference = −2.9 ± 0.9, p < 0.0011) contrasted to the dual therapy arm. Additionally, the rates of achieving the BP target (53.8% vs. 37.8%, p = 0.0017) as well as BP response (54.8% vs. 35.6%, p = 0.0001) were substantially greater in the triple therapy arm contrasted to the dual therapy arm. In both arms, there were no occurrences of drug discontinuation or treatment-related serious adverse events. Overall, 30 treatment-related adverse events were reported, with 20 (10.4%) and 10 (5.3%) occurring in the triple and dual therapy arms, respectively, without statistical significance (p = 0.0633). Lightheadedness was the most documented symptom in both arms, occurring in 11 (5.7%) patients in the triple therapy arm and four (2.1%) patients in the dual therapy arm. In conclusion, the findings indicated that a low-dose triple combination over eight weeks was safe and efficiently improved BP control in individuals with primary hypertension.

Discussion

In this systematic review, we evaluated the therapeutic efficacy of a triple therapy pill (amlodipine, telmisartan, and chlorthalidone) compared with various control groups, including usual care, high-dose amlodipine, or dual therapy of telmisartan and amlodipine, in patients with essential hypertension. Overall, the findings revealed that the triple therapy pill was associated with significant decreases in mean sitting systolic and diastolic BP, as well as greater frequencies of achieving BP control and response contrasted to the control arms. Additionally, the triple therapy pill was generally safe, with no considerable variation in the frequency of serious drug-related adverse events between the arms. While the data are encouraging, the results should be interpreted cautiously given the heterogeneity in drug doses, time points, and control groups.

Several RCTs have demonstrated that low-dose triple and quadruple combination therapies, using half or quarter doses, are effective pharmacological regimens for patients with hypertension [13,22-24]. Indeed, fixed low-dose combination therapy using affordable BP-lowering medications could effectively overcome various barriers to achieving better BP control. These combinations enhance efficacy [24,25], minimize adverse events at reduced doses [6], and provide additive benefits across different classes of antihypertensive medications [15]. Moreover, fixed-dose combinations simplify treatment regimens, potentially improving medication adherence [17,26] and alleviating challenges associated with frequent appointments and extended adjustment periods for patients, physicians, and health systems alike [27].

Triple therapy for lowering BP was initially introduced 50 years ago, and the concept of initiating low-dose triple treatment was first suggested in 2003 [6]. Evidence from RCTs has shown the benefits of triple therapy in patients with severe hypertension that is not sufficiently regulated with dual therapy [28]. Within these lines, Salam et al. examined 14 RCTs with close to 11,500 participants [28]. Overall, triple therapy compared to dual therapy resulted in a significant decrease in systolic BP by 5.4 mmHg and diastolic BP by 3.2 mmHg, and improved BP control rates (58% vs. 45%). The incidence of withdrawals secondary to harmful occurrences did not show significant differences between arms (3.3% vs. 3.4%) [28]. Therefore, adding a third pharmacological agent is expected to enhance efficacy without amplifying adverse events, contracted to upscaling the dose of an existing dual therapy regimen. Additionally, initiating triple treatment early can substantially enhance hypertension control. Notably, current global guidelines from the United States [9] and Europe [16] endorse triple, but not dual, therapy for the treatment of individuals with uncontrolled, resistant hypertension. The most frequently used triple therapy combination for blood pressure control includes ACEI/ARB, CCB, and thiazide/thiazide-like diuretics [9,10,16].

Amlodipine, a CCB, plays a pivotal role as a key component in triple antihypertensive therapy, offering a wide range of benefits as recently reviewed by Wang and colleagues [29]. For example, amlodipine exhibits low renal clearance, possesses a long half-life (35-50 hours), and displays a prolonged interval of activity, enabling sustained antihypertensive effects for over 24 hours after a single dose. Its efficacy includes maintaining BP control even in cases of missed doses and ensuring continuous protection against noncompliance. It is also effective in managing hypertension in patients with high systolic/diastolic BP, DM, or CKD without exacerbating glycemic or renal indices. Furthermore, amlodipine is particularly suitable for older adults owing to its competence to regulate BP and reduce the risk of stroke. Common side effects such as flushing and dizziness are more prevalent with higher doses, particularly 10 mg. Amlodipine is cost-effective and is expected to yield cost savings contrasted to standard care protocols [29].

ACEIs play crucial roles in first-line triple therapy for the management of hypertension for a multitude of benefits, as recently reviewed by Cutrell and partners [30]. Unlike ACEIs, ARBs do not inhibit ACE in the renin-angiotensin system, resulting in a lower risk of angioedema and cough. Recent findings also suggest potential neuroprotective effects of ARBs compared to other antihypertensive medications, including ACEIs, although additional research is needed to confirm this. Currently, both ACEIs and ARBs are equally endorsed as first-line therapies for managing hypertension. Recent evidence indicates that ARBs are as effective as ACEIs in treating hypertension but offer better tolerability [30].

The optimal choice of diuretic, i.e., hydrochlorothiazide or chlorthalidone, for managing hypertension has been a topic of argument for many years [31]. It is well established that chlorthalidone provides consistent BP control throughout the day [32] and is more potent with an extended period of antihypertensive activity contrasted to hydrochlorothiazide [33]. Moreover, recent studies have demonstrated that chlorthalidone is safe and effective for lowering BP in predialysis patients with stage 4 CKD [34]. Regarding side effects, there is evidence that chlorthalidone has a higher rate of hypokalemia compared to hydrochlorothiazide [35].

A post-hoc analysis of the TRIUMPH RCTs revealed that DM was a negative predictor of BP change, as patients with DM appeared to exhibit decreased efficacy of BP-lowering treatments contrasted to those without DM [36]. Lung et al. performed an economic valuation of the TRIUMPH trial [37]. In comparison to standard treatment, the triple-pill method cost an extra $9.63 per person in the within-trial analysis and $347.75 per person in the modeled analysis. The triple therapy was very cost-effective over the long run compared to standard treatment in Sri Lanka [37].

Our study presents the first systematic review of the safety and therapeutic utility of triple therapy pill (amlodipine, telmisartan, and chlorthalidone) from RCTs. However, our study is limited by a small sample size due to the low number of trials meeting our eligibility criteria. Additional limitations include the heterogeneity in control treatments, time points, and triple therapy doses, which could have impacted the confidence in our results. To elaborate, heterogeneity in drug doses, time points, and control groups can significantly affect the interpretation of results because it introduces variability that complicates comparisons and conclusions. Different drug doses can alter the effectiveness and side effects of treatments, leading to inconsistent outcomes across studies. Varying time points can influence how outcomes are measured, as the effects of treatment may differ over time. Additionally, differences in control groups can impact the baseline comparisons and the validity of the results. This variability makes it challenging to generalize findings and determine the true efficacy of the treatments, necessitating cautious interpretation of the data.

To overcome these limitations in future research, it is essential to standardize drug doses, treatment durations, and control group characteristics across studies to ensure consistency. Implementing well-defined protocols and using uniform measurement techniques can help reduce variability and enhance comparability. Additionally, conducting large-scale, multi-center trials with rigorous randomization and blinding procedures can improve the reliability of results. By addressing these factors, researchers can obtain more accurate and generalizable findings.

## Conclusions

The triple therapy pill (amlodipine, telmisartan, and chlorthalidone) was found to be safe and effective in patients with essential hypertension. This was demonstrated by significant reductions in mean sitting systolic and diastolic BP, as well as higher rates of achieving BP control and response compared to the control arms. Further research is needed, particularly focusing on long-term effects beyond six months. Other important outcomes to consider in future research include mean sitting diastolic BP and ambulatory BP measurements. Additionally, investigating the specific characteristics of patients that could serve as potential predictors, both positive and negative, of response to this therapy would be valuable.
